# Piperine ameliorates SCA17 neuropathology by reducing ER stress

**DOI:** 10.1186/s13024-018-0236-x

**Published:** 2018-01-30

**Authors:** Jifeng Guo, Yiting Cui, Qiong Liu, Yang Yang, Yujing Li, Ling Weng, Beisha Tang, Peng Jin, Xiao-Jiang Li, Su Yang, Shihua Li

**Affiliations:** 1 Department of Neurology and National Clinical Research Center for Geriatric Disorder, Xiangya Hospital, Central South University, Changsha, Hunan 410008 China; 2 Department of Human Genetics, Emory University School of Medicine, 615 Michael Street, Atlanta, GA 30322 USA; 3 GHM Institute of CNS Regeneration, Jinan University, Guangzhou, 510631 China

**Keywords:** Polyglutamine, Ataxia, Neurotrophic factor, ER stress

## Abstract

**Background:**

Spinocerebellar ataxia 17 (SCA17) belongs to the family of neurodegenerative diseases caused by polyglutamine (polyQ) expansion. In SCA17, polyQ expansion occurs in the TATA box binding protein (TBP) and leads to the misfolding of TBP and the preferential degeneration in the cerebellar Purkinje neurons. Currently there is no effective treatment for SCA17. Mesencephalic astrocyte-derived neurotrophic factor (MANF) is a recently identified neurotrophic factor, and increasing MANF expression ameliorated SCA17 neuropathology in TBP-105Q knock-in (KI) mouse model, indicating that MANF could be a therapeutic target for treating SCA17.

**Methods:**

In this study, we screened a collection of 2000 FDA-approved chemicals using a stable cell line expressing luciferase reporter, which is driven by MANF promoter. We identified several potential candidates that can induce the expression of MANF. Of these inducers, piperine is an agent that potently induces the luciferase expression or MANF expression.

**Results:**

Addition of piperine in both cellular and mouse models of SCA17 alleviated toxicity caused by mutant TBP. Although mutant TBP is primarily localized in the nuclei, the polyQ expansion in TBP is able to induce ER stress, suggesting that nuclear misfolded proteins can also elicit ER stress as cytoplasmic misfolded proteins do. Moreover, piperine plays its protective role by reducing toxicity caused by the ER stress.

**Conclusion:**

Our study established piperine as a MANF-based therapeutic agent for ER stress-related neuropathology in SCA17.

## Background

Polyglutamine (polyQ) diseases represent a class of genetically defined, late-onset neurodegenerative diseases, which are featured by selective neuronal vulnerability in distinct brain regions [1]. Spinocerebellar ataxia 17 (SCA17) is the latest addition to this disease family [2]. In SCA17, the polyQ expansion occurs in the TATA box-binding protein (TBP) [3], a well-known general transcription factor that plays important roles in mediating transcription by all three nuclear RNA polymerases [4, 5]. The normal range of polyQ number in human TBP is between 25 and 42, whereas SCA17 patients carry more than 46 repeats [3, 6]. Similar to other polyQ diseases, extremely long polyQ repeats (>62Q) in TBP lead to juvenile onset SCA17 [7]. SCA17 patients exhibit both motor and non-motor symptoms, which include ataxia, dystonia, cognitive impairments, psychiatric abnormalities, and seizures [8, 9]. Accordingly, pronounced degeneration in the cerebellum, accompanied by moderate and diffused cortical and brain stem atrophy, is typical in the brains of adult onset patients with SCA17 [3, 10]. Despite progresses in understanding the molecular mechanisms underlying SCA17 pathogenesis using various animal models [11–16], an effective treatment for SCA17 remains lacking.

Neurotrophic factors, such as BDNF, play important roles in mediating neuronal development, functions, and survival [17], and have long been studied as potential therapeutic agents for polyQ diseases [18–20]. Mesencephalic astrocyte-derived neurotrophic factor (MANF) represents a novel class of neurotrophic factors that are structurally and functionally distinct from these classical ones [21, 22]. Most uniquely, MANF possesses two modes of action: extracellularly, MANF potentially mediates signaling pathways, such as PKC signaling, through an unknown receptor [23], and administration of recombinant MANF protein is neuronal protective in a rat model of Parkinson’s disease [24]; intracellularly, MANF is abundantly expressed in the endoplasmic reticulum (ER), and functions as an ER-stress inducible protein [25, 26]. Emerging evidence suggests that MANF affords protection in several disease conditions, including Parkinson’s disease, ischemic stroke, and retinal degeneration [24, 27, 28]. MANF is also involved in the hypothalamic control of food intake activity [29]. Previously, we identified MANF as a target downregulated by TBP with polyQ expansion, and increasing MANF expression ameliorated SCA17 neuropathology in TBP-105Q knock-in (KI) mouse model [23], indicating that MANF is a potential therapeutic target for SCA17 treatment.

In the current study, we aimed to develop a MANF-based therapeutic strategy for SCA17. We screened a collection of 2000 US Food and Drug Administration (FDA) approved chemicals [30] in search for agents that induce MANF expression. We identified piperine as a potent MANF expression inducer, which, when added to the cell culture medium or given to mice via oral gavage, could significantly increase the level of MANF. Moreover, piperine treatment alleviated toxicity caused by mutant TBP, both in vitro and in vivo, by antagonizing ER stress. Our study established piperine as a promising drug for the treatment of SCA17 and perhaps other ER stress-related diseases.

## Methods

### Antibodies

The antibodies used in this study include: ATF6 (Novus Biologicals, NBP1-40256), MANF (LSBio, LS-B2688), XBP1s (BioLegend, 619502), GADD153/CHOP (Novus Biologicals, NB6000-1335), Calbindin (Millipore/Chemicon), 1TBP18 (QED bioscience, 70102), GAPDH, Actin (Sigma, A5060, 1:50,000). All secondary antibodies were purchased from Jackson Immunoresearch.

### Mouse lines and piperine treatment

The TBP-105Q germ-line KI mice and MANF transgenic mice were generated as described previously [23, 31]. We crossed germ-line TBP KI mice with MANF transgenic mice to generate the TBP KI/MANF mice. All mice were bred and maintained in the animal facility at Emory University under specific pathogen-free conditions in accordance with institutional guidelines of the Animal Care and Use Committee at Emory University.

For drug treatment, piperine (Sigma-Aldrich, P49007) was dissolved in saline to a final concentration of 80 mg/ml, and then administered to the mice daily via oral gavage at a dose of 10 mg/kg for two consecutive months.

### Mouse behavior tests

Mouse behavior tests were performed as described previously [32]. Briefly, rotarod test was done using an automated equipment (Rotamex, Columbus Instruments). Prior to the initial test, mice were trained on the rotarod at the speed of 5 rpm for 10 min for 3 consecutive days. During the test, the rotarod was set to accelerate from 0 rpm to 40 rpm, with an increment of 0.1 rpm per second. Each mouse was subjected to three trials, and the time it stayed on the rotarod was recorded automatically. The average time of three trials was used to evaluate the animal performance.

For balance beam test, mice were trained for 2 days to walk on a 0.6 cm wide × 80 cm long wooden beam that was suspended 50 cm above the floor. On each trial the mouse was released onto the end of the beam and required to run down the entire beam and into the dark box. Each mouse was tested, and each session was the average of 3 trials. The time for a mouse to cross to the end was recorded.

For grip strength test, mice were allowed to grip the metal grids of a grip meter (Ametek Chatillon) with all their limbs, and they were gently pulled backwards by the tail until they could no longer hold the grids. The peak grip strength observed in 5 trials was recorded.

### Cell culture

Stable TBP-13Q and TBP-105Q PC12 cell lines were generated in our previous study [33]. PC12 cells were cultured in Dulbecco’s modified Eagle’s medium (DMEM) supplemented with 10% horse serum, 5% fetal bovine serum, 100 U/ ml penicillin and 100 μg/ml streptomycin. N2a cells were cultured in DMEM supplemented with 10% fetal bovine serum, 100 U/ ml penicillin and 100 μg/ml streptomycin.

For piperine and tunicamycin (Sigma-Aldrich, T7765) treatment, we used DMEM supplemented with less serum (0.6% horse serum, 0.4% fetal bovine serum), 100 U/ ml penicillin and 100 μg/ml streptomycin. PC12 or N2a cells were cultured in normal culture medium for 24 h, and then the medium was replaced with the low-serum culture medium with or without piperine for 48 h. Tunicamycin was added to the culture medium for 4 h before cells were collected.

To reduce MANF expression in N2a cells, we used the CRISPR/Cas9 plasmids designed in our previous study [29]. The plasmids were transfected into N2a cells using lipofectamine 2000 (Thermo Fisher Scientific).

### Western blot, immunohistology and qRT-PCR

Methods for western blot and immunohistology were described previously [34]. Briefly, to prepare brain lysate, cortices and cerebellum tissues of the mice were dissected, homogenized in RIPA buffer with a glass homogenizer. Protein concentration was determined by BCA protein assay kit (Thermo Scientific, 23,227). For brain immunohistochemistry, mice were perfused with 4% PFA, fixed in 4% PFA for 24 h, followed by 30% sucrose buffer incubation until the brain sank to the bottom of the tube. Fixed brain tissue was embedded in O. C. T. compound (Fisher Healthcare), sectioned into 40 μm slices using a cryostat (Leica). Methods for RNA extraction and qRT-PCR were described previously [23]. The primers used are: MANF, forward: 5′ -ATG GAT CCA GGA TGT GGG CTA CGC -3′, reverse: 5′ -ATG AAT TCC AGA TCA GTC CGT GCG -3′; Actin, forward: 5′ -TGA GAC CTT CAA CAC CCC AG -3′, reverse: 5′ -GTG GTG GTG AAG CTG TAG CC- 3′.

### Luciferase reporter assay for drug screen

MANF luciferase reporter construct was generated in our previous study [23]. PC12 cells were transfected with MANF luciferase reporter using Lipofectamine 2000. Cells were selected with hyglomycine to isolate stably transfected PC12 cells expressing MANF luciferase reporter, which were used for drug screen. Drug screen was done in 96-well plates. All drugs used for screening were at 2.5 or 10 μM and added to the culture medium. After 48 h of treatment, the cells were collected and subjected to luciferase reporter assay using ONE-Glo Luciferase Assay System (Promega). Luciferase intensity was measured by Synergy H4 microplate reader.

### Statistical analysis

All values are expressed as mean ± SE. We assessed statistical significance using Student’s *t*-test and used ANOVA when multiple samples were compared. A *P*-value of less than 0.05 is considered to be significant.

## Results

### MANF overexpression ameliorated ER stress in TBP-105Q KI mice

**Fig. 1:**
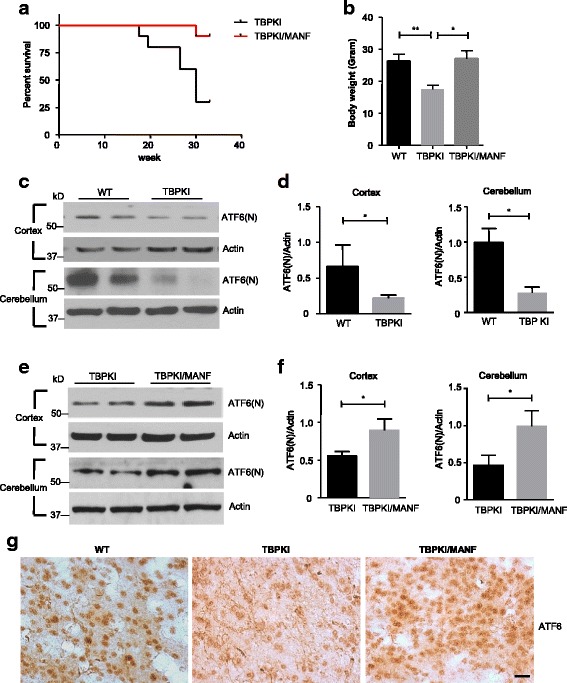
MANF overexpression extended lifespan and ameliorated ER stress in TBP KI mice. **a** Kaplan-Meier survival curve shows the lifespan of TBP KI and TBP KI/MANF mice (*n* = 10 for each phenotype, including 5 males and 5 females). **b** Mean body weight of 4-month-old WT, TBP KI and TBP KI/MANF mice (n = 10 for each phenotype, including 5 males and 5 females). * *P* < 0.05, ** *P* < 0.01. **c** Western blotting analysis of the expression of N-terminal ATF6 fragment (ATF6(N)) in the cortex and cerebellum of 4-month-old WT and TBP KI mice. Actin was used as a loading control. **d** Quantification of the ratio of ATF6(N) to actin on western blots in Fig. 1c (*n* = 3, * *P* < 0.05). **e** Western blotting analysis of the expression of ATF6(N) in the cortex and cerebellum of 4-month-old TBP KI and TBP KI/MANF mice. **f** Quantification of the ratio of ATF6(N) to actin on western blots in Fig. 1e (*n* = 3, * *P* < 0.05). **g** Immunohistochemistry staining of ATF6 in the cortex of 4-month-old WT, TBP KI and TBP KI/MANF mice (Scale bar: 20 μm)

In our previous study, we generated a transgenic mouse model that overexpresses MANF in the central nervous system. By crossing MANF transgenic mice with TBP-105Q KI mice, we found that MANF overexpression alleviated motor impairments in TBP-105Q KI mice [23]. Here we extended the scope of our previous study to evaluate the influence of MANF overexpression on life span of TBP-105Q KI mice. Mutant TBP toxicity led to premature lethality in TBP-105Q KI mice, as death could be observed as early as 18 weeks, and more than 70% of mice died within 30 weeks. In contrast, TBP-105Q KI mice with MANF overexpression exhibited significantly extended life span, as evidenced by more than 90% of mice that had survived past 33 weeks (Fig. 1a). Another pathological feature of TBP-105Q KI mice is loss of body weight. At 4-months of age, TBP-105Q KI mice showed significantly reduced body weight, whereas TBP-105Q KI/MANF mice had comparable body weight to wild type (WT) littermates at 4 months of age (Fig. 1b). The additional evidence for significantly prolonging the life span of TBP-105Q KI mice supports the protective effect of MANF on the neuropathology in SCA17 mice as reported in our previous study [23].

It is well established that the expression of MANF is induced upon ER stress, and MANF functions as a protective factor in ER stress-induced cell death [26, 27, 35, 36]. We want to ascertain if MANF affords neuroprotection by mitigating ER stress in TBP-105Q KI mice. ATF6 is a pro-survival transcription factor and is reported to be induced in the cortex of the brain by different types of ER stresses [37–39]. Since the upregulation of cortical ATF6 represents an ER stress response, we examined the ATF6 level in the cortex and cerebellum of TBP-105Q KI mice using western blotting analysis with anti-ATF6 antibody. We found significantly reduced expression of N-terminal ATF6, the biologically active form of ATF6, in both the cortex and cerebellum of TBP-105Q KI mice compared with WT (Fig. 1c, d), suggesting that mutant TBP-105Q has induced ER stress in the TBP KI mouse brain and that the brain expressing TBP-105Q may be more vulnerable to damages caused by additional ER stress. Nonetheless, MANF overexpression significantly increased the level of N-terminal ATF6 in both the cortex and cerebellum of TBP-105Q KI mice (Fig. 1e, f). Immunocytochemical analysis also showed that ATF6 staining was decreased in the cortex of 4-month-old TBP-105Q KI mice and that this decrease was reversed by expression of transgenic MANF (Fig. 1g). Therefore, MANF potentially ameliorates mutant TBP toxicity by suppressing ER stress.

### Large-scale screening for MANF inducers

**Fig. 2:**
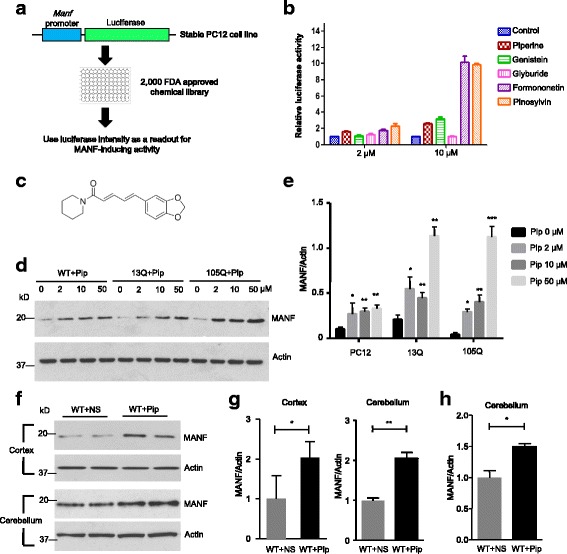
Large-scale drug screening identified piperine as a MANF expression inducing agent. **a** Schematic representation of the design for large-scale drug screening. **b** Luciferase intensity of stable PC12 cells treated with different drugs (Piperine, Genistein, Glyburide, Formononetin and Pinosylvin) at different concentrations (2 μM and 10 μM). Cells treated with DMSO only were used as a control (n = 3). **c** Chemical structure of piperine. **d** Western blotting analysis of WT, TBP-13Q, and TBP-105Q PC12 cells treated with different concentrations (2 μM, 10 μM and 50 μM) of piperine for 48 h. Cells treated with DMSO (0 μM) only were used as a control. Actin was used as a loading control. **e** Quantification of the ratio of MANF to actin on western blots in Fig. 2d (n = 3, * *P* < 0.05, ** *P* < 0.01, *** *P* < 0.001). **f** Western blotting analysis of MANF expression in the cortex and cerebellum of WT mice after 2-month treatment with either saline (WT + NS) or piperine (10 mg/kg, WT + Pip). Four mice per group were used for analysis. **g** Quantification of western blotting result in Fig. 2f (*n* = 4, * *P* < 0.05, ** *P* < 0.01). **h** qRT-PCR analysis of MANF mRNA level in the cerebellum of WT mice after 2-month treatment with either saline (WT + NS) or piperine (10 mg/kg, WT + Pip) (n = 3, * *P* < 0.05). Results are presented as mean ± SEM

The neuroprotective effects of MANF in TBP-105Q KI mice prompted us to search for chemicals that induce MANF expression as potential therapeutics for SCA17. To look for such candidates from a library of 2000 FDA approved chemicals, we generated a stable PC12 cell line expressing a luciferase reporter under the control of a 300-bp MANF promoter [23]. The cells were treated with 2.5 or 10 μM of each chemical for 48 h, and the luciferase intensity produced in PC12 cells was measured by a luciferase reader, and the value of the luciferase units were used as readout for MANF-inducing potency (Fig. 2a). This strategy allowed us to identify several chemicals that dramatically increased luciferase production in stable transfected PC12 cells, including piperine, genistein, glyburide, formononetin and pinosylvin (Fig. 2b). Among these chemicals, genistein and formononetin share a similar chemical structure, whereas others are structurally different. We focused our attention on piperine (C_17_H_19_NO_3_, Fig. 2c), as its anti-inflammatory effect has been well established [40–42]. Moreover, the low cost of piperine makes it possible to test its neuroprotective effect in mice.

To further test the effect of piperine, we treated WT PC12 cells as well as stable transfected PC12 cell expressing either TBP-13Q or TBP-105Q [33] with different concentrations of piperine. We found that MANF protein level was indeed increased in a dose-dependent manner after piperine treatment (Fig. 2d, e). Since piperine can increase MANF production in the PC12 cell model, we treated WT mice with 10 mg/kg body weight of piperine by daily oral gavage to determine if piperine can stimulate MANF expression in vivo. After two-month treatment, the cortex and cerebellum from WT mice treated with piperine showed significantly increased expression of MANF, compared with the brains of mice treated with saline (Fig. 2f, g). In addition, qRT-PCR analysis confirmed the increased MANF mRNA level in the cerebellum of piperine-treated mice (Fig. 2h). These results confirmed the validity of our large-scale screening, and also provided us confidence to test the protective potential of piperine in SCA17 mouse models.

### Piperine treatment reduced ER stress in vitro

**Fig. 3:**
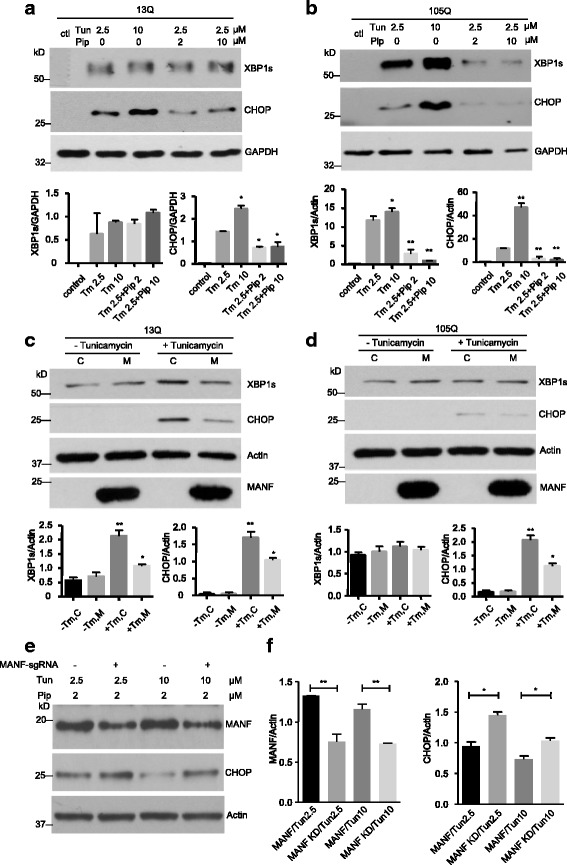
Piperine reduced ER stress caused by mutant TBP. **a**, **b** Stable transfected PC12 cells expressing TBP-13Q (**a**) or TBP-105Q (**b**) were treated with different concentrations (0, 2 or 10 μM) of piperine for 48 h. Different concentrations of tunicamycin (0, 2.5 or 10 μM) were then added for 4 h. Western blotting was performed to examine the levels of XBP1s and CHOP. Untreated cells (ctl) were used as controls. GAPDH was used as a loading control. The ratios of XBP1s or CHOP to GAPDH are presented beneath the blots (n = 3, * *P* < 0.05). **c**, **d** PC12 cells expressing TBP-13Q (**c**) or TBP-105Q (**d**) were transfected with either MANF (M) or empty vector (C), followed by treatment with 2.5 μM of tunicamycin for 4 h. Western blotting was performed to check the levels of XBP1s and CHOP. Actin was used as a loading control. Quantification of the ratios of XBP1s or CHOP to actin is presented beneath the blots (*n* = 3, * *P* < 0.05, ** *P* < 0.01). **e** N2a cells were transfected with either MANF-sgRNA and Cas9 (MANF-sgRNA +), or control-sgRNA and Cas9 (MANF-sgRNA -), treated with 2 μM of piperine for 48 h. Different concentrations of tunicamycin (2.5 or 10 μM) were then added for 4 h. Western blotting was performed to examine the expression of MANF and CHOP. Actin was used as a loading control. **f** Quantification of western blotting result in Fig. 3e. MANF KD (knockdown) was used to indicate cells transfected with MANF-sgRNA and Cas9 (n = 3, * *P* < 0.05, ** *P* < 0.01). Results are presented as mean ± SEM

To test if piperine can protect mutant TBP-mediated toxicity, we first checked the protective effect of piperine in a cellular model of SCA17. Tunicamycin is a commonly used ER stress inducer [43, 44]. Treating stable TBP-13Q- or TBP-105Q-PC12 cells with tunicamycin for 4 h induced ER stress, as evidenced by the increased levels of XBP1s and CHOP, both of which are transcriptional factors that are activated by ER stress [45–48]. Intriguingly, in PC12 cells without piperine treatment, there was a great induction of ER stress by tunicamycin, as evidenced by marked increases in XBP1s and CHOP (Fig. 3a, b). These increases were suppressed by piperine, as both XBP1s and CHOP levels were significantly reduced, and the extent of suppression appeared to be more dramatic in PC12 cells expressing TBP-105Q (Fig. 3a, b), which suggest that mutant TBP may exacerbate ER stress induced by tunicamycin, whereas piperine effectively counteracted the toxicity caused by mutant TBP.

Since piperine treatment induced MANF expression in PC12 cells and produced protective effect, we wanted to explore if overexpression of MANF in the PC12 cell could reproduce the protective effect of piperine. We transfected the plasmid encoding HA tagged MANF into PC12 cells that stably express either TBP-13Q or TBP-105Q. Indeed, transfected MANF was able to ameliorate ER stress induced by tunicamycin treatment (Fig. 3c, d). On the other hand, we utilized our recently established CRISPR/Cas9 constructs [29] to reduce the expression of MANF in N2a cells, which abolished the protective effect of piperine against tunicamycin-induced increase in CHOP or toxicity (Fig. 3e, f). Thus, our result suggests that piperine may antagonize ER stress by activating MANF expression.

### Piperine treatment ameliorated SCA17 neuropathology by suppressing ER stress

**Fig. 4:**
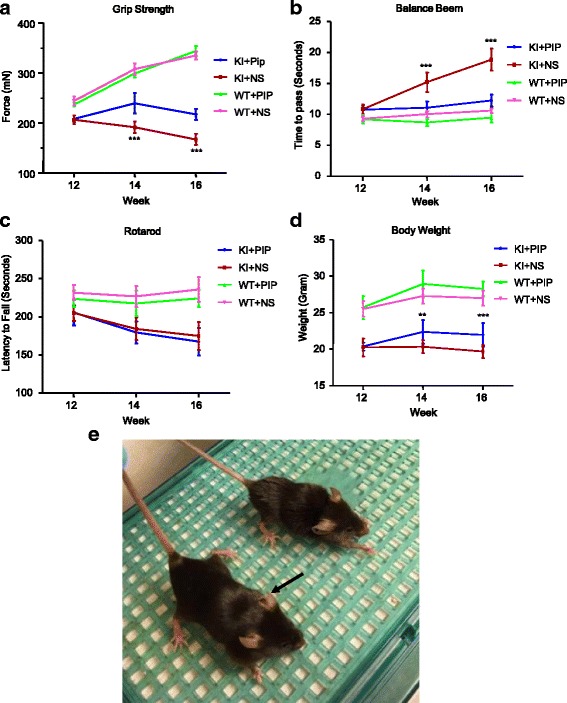
Piperine treatment improved motor performances of TBP-105Q KI mice. Three-month old WT and TBP-105Q KI mice were treated with either piperine (10 mg/kg, WT + Pip and TBP + Pip) or saline (WT + NS and TBP + NS) via daily oral gavage for 2 months (n = 10 for each group). Different behavioral tests were performed every 2 weeks. **a**-**d** TBP-105Q KI mice treated with piperine showed significant improvement in grip strength (**a**) and balance beam (**b**) tests compared with TBP-105Q KI mice treated with saline. TBP-105Q KI mice treated with piperine showed comparable rotarod performances as TBP-105Q KI mice treated with saline (**c**). The body weight of TBP-105Q KI mice treated with piperine was increased compared with TBP-105Q KI mice treated with saline (**d**). ** *P* < 0.01, *** *P* < 0.001. Results are presented as means ± SEM. **e** A representative image of 5-month-old TBP-105Q KI mice after 2 months of treatment with either piperine or saline. The black arrow indicates the mice treated with piperine

Next, we evaluated the protective effect of piperine using the mouse model of SCA17. In TBP-105Q KI mice, neuropathological phenotypes were initially observed around 3 months of age [31]. Therefore, we started piperine treatment in 3-month-old TBP-105Q KI mice by daily oral gavage, and recorded their motor performance before and after the treatment. At 3 months of age, TBP-105Q KI mice exhibited muscle atrophy and reduced muscle strength [31]. However,piperine treatment improved muscle function in TBP-105Q KI mice, as demonstrated by increased grip strength. TBP-105Q KI mice also had impaired motor function evidenced by poor performance in balance beam and rotarod tests. Piperine treatment was able to restore the deficit in balance beam test, but not in rotarod test (Fig. 4a). As balance beam test is more sensitive than rotarod in determining motor coordination function [49], peperine may have more protective effects on those neuronal cells that control fine motor coordination. Moreover, piperine treatment partially alleviated body weight loss of TBP-105Q KI mice (Fig. 4a, b).

**Fig. 5:**
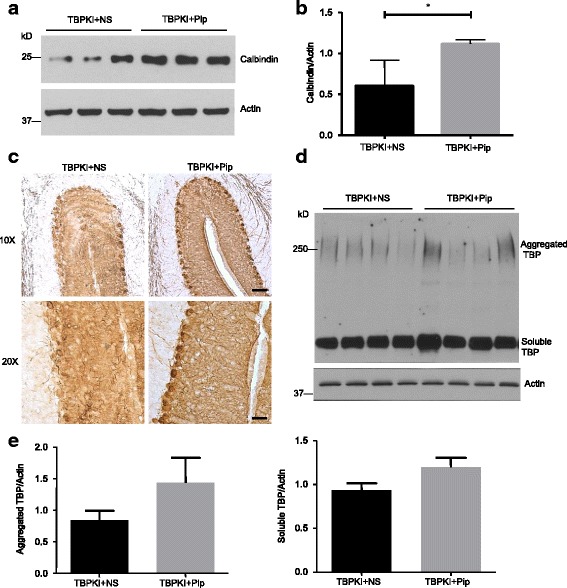
Piperine treatment ameliorated Purkinje cell degeneration in TBP-105Q KI mice. **a** Western blotting analysis of calbindin levels in the cerebellum of TBP-105Q KI mice treated with saline (TBP + NS) or piperine (TBP + Pip). Actin was used as a loading control. **b** Quantification of the ratios of calbindin to actin on western blots in Fig. 5a (n = 3, * *P* < 0.05). **c** Immunohistochemistry staining of calbindin in the cerebellum of TBP-105Q KI mice treated with saline or piperine (Scale bar: left, 50 μm; right, 20 μm). **d** Western blotting analysis of mutant TBP in the cerebellum of TBP KI mice treated with saline or piperine. Four mice per group were examined. Aggregated and soluble forms of TBP are indicated. **e** Quantification of western blotting result in Fig. 5d (*n* = 4). Results are presented as means ± SEM

To examine if piperine treatment could improve pathological event in SCA17 mice, we collected the brains of the SCA17 mice at the end of the treatment. A typical pathological hallmark of SCA17 patients is Purkinje cell degeneration in the cerebellum [3, 10], which has been successfully recapitulated in several SCA17 mouse models, including TBP-105Q KI mice [15, 23, 50]. We compared the level of calbindin, a Purkinje cell marker, in the cerebellum of TBP-105Q KI mice treated with saline or piperine. Remarkably, TBP-105Q KI mice treated with piperine showed significantly higher level of calbindin staining (Fig. 5a, b), indicating improved Purkinje cell survival. This result is further corroborated by the immunohistochemical study, as TBP-105Q KI mice treated with piperine displayed a more intact structure of Purkinje cells compared with mice treated with saline (Fig. 5c). Taken together, these results confirmed the protective effect of piperine treatment against SCA17 neuropathology. Nonetheless, we found no significant changes in either the aggregated or soluble forms of mutant TBP in the treated TBP-105Q KI mice (Fig. 5d, e), which suggests that piperine exerts its protection through modulating certain cellular functions that might have been affected by mutant TBP, rather than directly decreases the expression of mutant TBP.

**Fig. 6:**
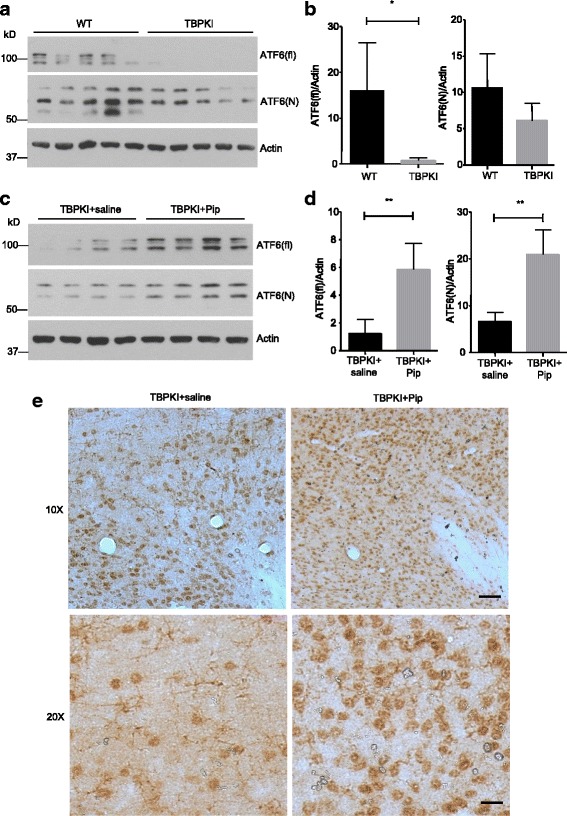
Piperine treatment increased ATF6 expression in TBP-105Q KI mice. **a** Western blotting analysis of full length (ATF6(fl)) and N-terminal ATF6 (ATF6(N)) in the cortex of WT and TBP-105Q KI (TBP KI) mice at 6 months of age. Five mice per group were analyzed. **b** Quantification of the ratios of ATF6(fl) or ATF6(N) to actin on western blots shown in Fig. 6a (*n* = 5, * *P* < 0.05). **c** Western blotting analysis of full length and N-terminal ATF6 in the cortex of TBP-105Q KI mice treated with either saline (TBP KI + saline) or piperine (TBP KI + Pip) for 8 weeks. Four mice per group were used for analysis. **d** Quantification of the ratios of ATF6(fl) or ATF6(N) to actin on western blots shown in Fig. 6c (*n* = 4, ** *P* < 0.01). Results are presented as means ± SEM. **e** Immunohistochemistry staining of ATF6 in the brain of TBP-105Q KI mice treated with either saline or piperine for 8 weeks (Scale bars: upper, 50 μm; lower, 20 μm)

If piperine treatment ameliorated ER stress to execute its protective function, we expect that piperine treatment could reduce ER stress, which should be reflected by restored ATF6 levels. Because the upregulation of ATF6 in the brain cortex is found to antagonize ER stress [37–39], which could be evaluated via western blotting, we collected mouse brain cortex tissues at the end of piperine treatment and examined ATF6 levels with western blotting. We found that the reduction of both full-length and N-terminal ATF6 in TBP-105Q KI mice treated with saline control (Fig. 6a, b). However, the TBP-105Q KI littermates treated with piperine showed a significant increase in both full-length and N-terminal ATF6 (Fig. 6c, d). This finding was further supported by immunohistochemical studies, as a more intense staining of ATF6 was found in the brains of TBP-105Q KI mice treated with piperine, compared with those treated with saline control (Fig. 6e). Taken together, piperine treatment is likely to execute its protective effects by reducing ER stress.

## Discussion

There are two important findings in our current studies. First, by screening 2000 FDA-approved chemicals, we identified piperine as a neuronal protective compound via regulating MANF expression. Second, we found that ER stress is involved in the neuropathology in SCA17 mice, which could be reduced by piperine treatment.

ER stress arises when excessive unfolded or misfolded proteins accumulate in the lumen of ER. Upon ER stress, several cellular mechanisms are activated to restore normal cellular functions, which include halting protein synthesis, promoting protein refolding and degradation. Extended ER stress triggers self-destruction signals, which lead to apoptosis [44, 51]. Given the close relationship between ER stress and protein homeostasis, remarkable efforts have been devoted to study the involvement of ER stress in neurodegenerative proteinopathies. Indeed, emerging evidence supports that ER stress is a common pathological event underlying the development of major neurodegenerative diseases, including Alzheimer’s disease (AD), Parkinson’s disease (PD), amyotrophic lateral sclerosis (ALS), and Huntington’s disease (HD) [52–56]. In these diseases, misfolded proteins are largely expressed in the cytoplasm. In the present study, we found that ER stress also potentially contributes to the pathogenesis of SCA17 that is primarily caused by misfolded proteins in the nuclei. Specifically, mutant TBP with polyQ expansion decreased the expression of ATF6, a transcription factor known to play a protective role in ER stress [35, 57, 58]. This is in agreement with our previous studies, as microarray analysis using mutant TBP transgenic mouse brain revealed expression changes of several molecules involved in ER stress [50]. Our findings suggest that ER stress is also involved in the pathologic event in SCA17 and that mutant misfolding proteins can mediate neurodegeneration through ER stress regardless of the different functions and subcellular localization of the disease proteins.

The evidence to support the involvement of ER stress in SCA17 is also derived from the fact that MANF shows protective effects on SCA17 neuropathology in TBP-105Q KI mouse model [23]. A unique feature of MANF is that it possesses biological functions both extracellularly and intracellularly. Extracellularly, addition of MANF protects specific types of neurons in vitro and in vivo [24, 59]. Although the receptor for MANF remains unknown, MANF could trigger PKC activation, which potentially serves as a pro-survival signal in SCA17 [23]. On the other hand, more attention has been focused on the intracellular function of MANF, as a plethora of studies indicate that MANF is localized in the ER and protects against ER stress- induced cell death [25, 27, 60, 61]. Our current study finds that MANF ameliorates mutant TBP toxicity by reducing ER stress. Therefore, the protective mechanism of MANF in SCA17 is likely two-fold, by mediating pro-survival signals outside the cell, and by maintaining protein homeostasis in the ER. We recently reported that immunoactivation in glia contributes to neurotoxicity in TBP-105Q KI mice [16]. Given that MANF is able to modulate inflammatory response in glia [28], it is likely that at least part of the neuroprotective effect of MANF is from non-cell-autonomous pathways.

In an effort to develop the MANF-based therapy, we screened a library of 2000 chemicals, all of which are FDA approved. Therefore, the result of our screening is of immediate value to the development of SCA17 treatment. Among a handful of positive chemicals after the screening process, we focused on piperine, which is a well established anti-inflammatory agent [41, 42, 62, 63]. It should be noted that inflammation is also a major cellular response induced by ER stress [64–66], which provides additional credence to the potential use of piperine to treat SCA17. The new finding in our study is that piperine can induce MANF expression to execute its anti-ER stress effect. Also, we found that piperine treatment ameliorated ER stress induced by tunicamycin in culture cells. Although we have evidence from the cultured cells and mouse brains to support the anti-ER stress effect of piperine, it is important to explore whether piperine is protective against the SCA17 neuropathology, which is featured by Purkinje cell degeneration that can be assessed by immunocytochemistry. Indeed, oral administration of piperine to the mice increased MANF expression in the brain, and also alleviated SCA17 neuropathology caused by mutant TBP. Previous studies have shown that piperine is protective in various Alzheimer’s disease models, and the protective efficacy appears to involve anti-inflammation, anti-oxidation and anti-excitotoxicity [67–70]. Therefore, although our data indicates that MANF expression is required for piperine’s function in vitro, we cannot rule out the possibility that other mechanisms beyond the upregulation of MANF also contribute to the protective effects of piperine in SCA17 mouse model. Nonetheless, both piperine treatment and transgenic MANF overexpression were able to increase the expression of ATF6, indicating that the MANF-inducing effect represents an important aspect of piperine’s therapeutic capacity.

## Conclusion

Altogether, our study identified ER stress as an integral part of SCA17 pathogenesis, and established piperine as a promising MANF-based therapy for treating SCA17, which also has therapeutic implications for other ER stress associated neurodegenerative diseases.

## Abbreviations

ER: Endoplasmic reticulum
KI: Knock-in
MANF: Mesencephalic astrocyte-derived neurotrophic factor
polyQ: Polyglutamine
SCA17: Spinocerebellar ataxia 17
TBP: TATA-box binding protein
